# Exploration of Damage Identification Method for a Large-Span Timber Lattice Shell Structure in Taiyuan Botanical Garden based on Structural Health Monitoring

**DOI:** 10.3390/s23156710

**Published:** 2023-07-27

**Authors:** Guoqing Wang, Chenjia Xu, Shujia Zhang, Zichun Zhou, Liang Zhang, Bin Qiu, Jia Wan, Honggang Lei

**Affiliations:** College of Civil Engineering, Taiyuan University of Technology, Taiyuan 030024, China; wangguoqing0066@link.tyut.edu.cn (G.W.); xuchenjia0072@link.tyut.edu.cn (C.X.); zhangshujia0076@link.tyut.edu.cn (S.Z.); zhouzichun0049@link.tyut.edu.cn (Z.Z.); zhangliang0073@link.tyut.edu.cn (L.Z.); qiubin@tyut.edu.cn (B.Q.); wanjia@tyut.edu.cn (J.W.)

**Keywords:** damage identification, damage sensitive feature, spatial lattice structure, structural health monitoring, timber structure, time series model

## Abstract

Large-span spatial lattice structures generally have characteristics such as incomplete modal information, high modal density, and high degrees of freedom. To address the problem of misjudgment in the damage detection of large-span spatial structures caused by these characteristics, this paper proposed a damage identification method based on time series models. Firstly, the order of the autoregressive moving average (ARMA) model was selected based on the Akaike information criterion (AIC). Then, the long autoregressive method was used to estimate the parameters of the ARMA model and extract the residual sequence of the autocorrelation part of the model. Furthermore, principal component analysis (PCA) was introduced to reduce the dimensionality of the model while retaining the characteristic values. Finally, the Mahalanobis distance (MD) was used to construct the damage sensitive feature (DSF). The dome of Taiyuan Botanical Garden in China is one of the largest non-triangular timber lattice shells worldwide. Relying on the structural health monitoring (SHM) project of this structure, this paper verified the effectiveness of the damage identification model through numerical simulation and determined the damage degree of the dome structure through SHM measurement data. The results demonstrated that the proposed damage identification method can effectively identify the damage of large-span timber lattice structures, locate the damage position, and estimate the degree of damage. The constructed DSF had relatively strong robustness to small damage and environmental noise and has practical application value for SHM in engineering.

## 1. Introduction

During long-term service, structures may suffer damage due to material aging, corrosion, prolonged loading, and natural disasters such as fire and earthquakes [1]. For major engineering structures, such as large-span bridges, high-rise buildings, large-span spatial lattice structures, large-scale water conservancy structures, and large-scale offshore platforms, failure can cause significant economic damage and human casualties. Therefore, SHM is of great significance in civil engineering and related fields [2].

Damage identification and localization are crucial processes in SHM since damage alters the stiffness, mass, or damping of structures. Consequently, stiffness reduction caused by cracking or loosening of connections will lead to changes in structural vibration modes, resulting in alterations in its dynamic response [3,4]. However, obtaining complete and accurate high-order modal information through theoretical analysis for actual structures, particularly for large-span spatial structures with numerous degrees of freedom, is challenging due to the complex vibration data involved. In addition, in practical engineering, it is almost impossible to obtain input and output signals by artificially exciting the structure and accurately identifying the structural modal information.

The damage identification method based on time series does not involve modal parameter identification. This method is based on statistical theory and can represent a large amount of effective information contained in structural response data with fewer parameters. By identifying the change pattern of the system through the interrelationships within the data, the changes in the model parameters can be used as the basis for detecting the existence and location of damage [5]. The time series model is sensitive to the identification of small structural damage, with strong noise reduction ability and operability, and has great potential in theoretical research and engineering applications. In recent years, it has received widespread attention and development from scholars [6,7].

The ARMA model, as an important component of time domain methods, is often used to fit regression structures to acceleration response data and extract DSF from it. Zuo and Guo [8] proposed a nonlinear damage identification method based on the autoregressive (AR) model and Kullback–Leibler distance, which has high sensitivity to minor damage. Razavi et al. [4]. suggested a damage feature identification method based on ARMA model and residual sample power spectral density, introducing Jeffrey distance and Smith distance for damage localization, and verifying the effectiveness of the damage identification model through vibration response experiments. Zhu et al. [9] studied the correlation between AR coefficients and structural stiffness reduction, proposing a method to identify structural damage using underdetermined equations established by AR coefficients, and solving underdetermined equations using the sparse regularization method. The effectiveness of the method was verified through experiments. Chen et al. [10] proposed a method for identifying structural nonlinear damage based on ARMA model and vector space cosine similarity (VSCS), and verified the feasibility of the method through experimental studies, solving the nonlinear problems that traditional methods cannot effectively handle caused by structural damage. Zeng et al. [11] advanced a time series model based on fuzzy c-means clustering algorithm, characterizing the degree of structural damage by the change of model coefficients, and verifying the feasibility and accuracy of the method through experimental and numerical studies. The method has low computational cost and is suitable for real-time monitoring of civil engineering. Diao et al. [12] combined the AR model with cointegration in econometrics to cointegrate variables at different nodes of the offshore platform. The method employs cointegration residuals as damage indicators and utilizes X-bar control charts for structural damage identification. The effectiveness of this approach was validated through both numerical simulation and experimental studies. Hu et al. [13] discussed a nonlinear ARMA(*n*,*m*) time series structural damage assessment method based on residual algorithm, proposing that higher-order models are more sensitive to disguised outliers, and verifying the method through dynamic monitoring tests. Liu et al. [7] explored the implementation method of damage feature extraction and damage warning for structures based on the ARMA model, and verified the significant changes of DSF index before and after damage through a *t*-test.

After obtaining the DSF, structural damage identification is reduced to a pattern recognition problem. Machine learning, as the main method of pattern recognition, includes Gaussian processes, support vector machines, neural networks, etc. Tang et al. [14] presented a damage identification method based on the AR model and Gaussian process, introducing parameters that characterize damage location and state information, and achieving a probability output of multi-damage localization and damage severity. Sui et al. [15] obtained the feature vector by arranging the damage indicators obtained by the AR model in order, and inputting it into the support vector machine optimized by a Bayesian algorithm for structural damage identification. The effectiveness of the method in identifying structural damage location was verified through experiments. Xu et al. [16] proposed a damage identification method based on the AR model and a BP neural network for lattice shell structures, which does not require modal parameter identification and excitation information, avoids the dependence of damage identification results on the accuracy of structural finite element (FE) models, and has high damage identification accuracy.

This article introduced PCA on the basis of existing research to extract features from the residual sequence. The advantage of incorporating PCA lies in its ability to reduce the dimensionality of the data while retaining the most informative features [17]. This not only simplifies the subsequent analysis but also helps to identify the most significant damage indicators. In addition, the MD was used in constructing DSF because it takes into account the differences and correlations in the variability of each observed variable. In addition, it can effectively eliminate the influence of scale on different measurement values.

Furthermore, existing studies were limited to theoretical research and simple experimental verification, lacking engineering applications and practices for large and complex structures. Based on the SHM project for a timber lattice shell dome in Taiyuan Botanical Garden, this paper proposes a damage identification method based on time series analysis. The effectiveness of the damage identification model was verified using numerical simulation, and the degree of damage to the lattice shell structure was identified using measured data from SHM. This paper combined theoretical research with engineering applications to verify the effectiveness and feasibility of applying the time-series-analysis-based damage identification method for practical engineering.

## 2. Research Background

**Figure 1:**
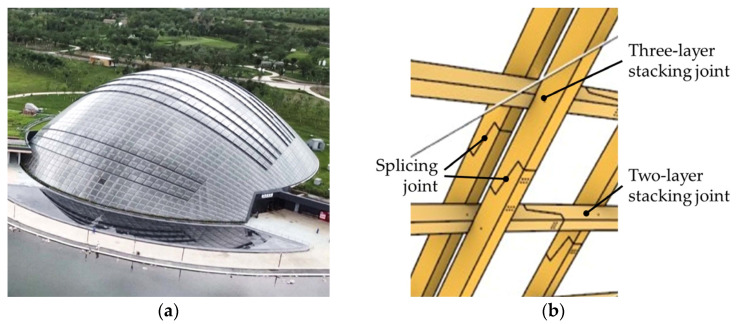

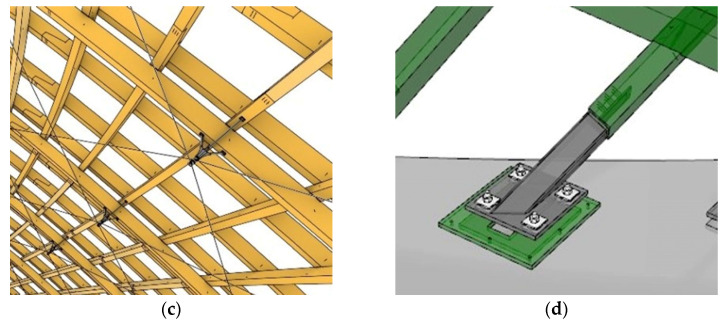
Timber lattice shell dome of Taiyuan Botanical Garden. (**a**) Appearance; (**b**) joint and its spatial relationship; (**c**) bidirectional cross steel cable; (**d**) root support.

This study took the timber lattice shell of Taiyuan Botanical Garden as the research background, which is located in Taiyuan, Shanxi, China. It is one of the largest non-triangular timber lattice structures worldwide. It was designed by Delugan Meissl Associated Architects (DMMA) from Austria and is now a landmark building in Taiyuan. The dome, from above, is shaped like a shell and glazed with double-curved panes of glass. The span of the dome is 89.5 m, with a span-to-height ratio of 3.0 and a projected area of approximately 6000 m^2^. The dome consists of double-curved laminated timber beams, which are arranged in two or three intersecting layers. The beams in the intersection joint area are connected by steel pins and self-tapping screws. The splicing connection of the beam adopts the half-lap joint form and is connected by self-tapping screws. To improve the overall rigidity of the structure, a bidirectional prestressed steel cable net was installed inside it, arranged diagonally to the timber beams. The steel support of the dome is connected to the timber beam using anti-shear steel plates and self-tapping screws and connected to the concrete foundation using chemical anchor bolts. The appearance and structural details of the dome are shown in Figure 1. For more details of the dome, please refer to reference [18].

## 3. Methodology

This section provides an overview of the fundamental principles of utilizing the ARMA model for time series analysis. It also elucidates the techniques for determining the order and estimating the parameters of the ARMA model using AIC and LAR. Furthermore, this section offers a theoretical exposition of the principal component analysis method introduced and defines a DSF construction method based on MD.

### 3.1. Time Series Analysis Modeling

Time series analysis is a statistical method for dynamic data processing, which is based on the theory of stochastic processes and mathematical statistics. It uses parameter models to process ordered random sampling data and studies the statistical laws followed by data sequences, in order to perform system identification [19,20]. Any structure can be regarded as a mechanical system composed of stiffness, mass, and damping matrices, which can be described by a motion differential equation and can be transformed into a difference equation in the discrete time domain. This difference equation has the same expression form as the time series model. Therefore, the equivalent relationship between the time series model and the structural motion differential equation can be used to determine the state of the structure and perform damage identification of the system.

#### 3.1.1. Basic Theory of ARMA Model

The ARMA model is a statistical model commonly used in time series analysis. It takes past observations of the time series as independent variables and current observations as the dependent variables to describe the randomness and autocorrelation of the time series data. The ARMA model consists of two parts: the AR model and the moving average (MA) model, and has the characteristics of both models. The AR or MA model is a special case of the ARMA model, so the ARMA model is more general for system response and is the most commonly used model in time series analysis.

For a multi-degree-of-freedom system, its vibration differential equation is:

(1) $$M\ddot{x}\left(t\right)+C\dot{x}\left(t\right)+Kx\left(t\right)=F(t),$$

where *M*, *C*, and *K* represent the mass matrix, damping matrix, and stiffness matrix of the structure, respectively. $\ddot{x}(t)$, $\dot{x}(t)$, and x(t) represent the acceleration vector, velocity vector, and displacement vector of the structure, respectively. F(t) is the excitation force applied to the system.

Equation (1) is equivalent to a 2*n*-order non-homogeneous differential equation system. Under the action of a single excitation f(t), the vibration differential equation is expressed as follows:

(2) $$\begin{array}{c} \varphi_{2n}x^{\left(2n\right)}+\varphi_{2n-1}x^{\left(2n-1\right)}+\ldots +\varphi_{1}\dot{x}+\varphi_{0}x\\ =\theta_{2n-2}f_{t}^{\left(2n-2\right)}+\theta_{2n-1}f_{t}^{\left(2n-1\right)}+\ldots +\theta_{1}\dot{f_{t}}+\theta_{0}f_{t} \end{array}$$

Discretizing Equation (2) and using a white noise sequence $a_{t}\sim ND({0,\sigma }_{a}^{2}$) as the system input yields a single degree of freedom ARMA (*p*,*q*) model [21]:

(3) $$x_{t}-\sum_{i=1}^{p}\varphi_{i}x_{t-i}=\theta_{0}a_{t}-\sum_{j=1}^{q}\theta_{j}a_{t-j}$$

In the equation, $\varphi_{i}$ represents the *i*-th order AR coefficient, $\theta_{j}$ represents the *j*-th order MA coefficient, and *p* and *q* are the AR and MA orders, respectively. By introducing a backward shift operator *B*, defined as $B^{k}x_{t}=x_{t-k}$ [5], Equation (3) can be transformed into:

(4) $$x_{t}=\frac{1-\sum_{j=1}^{q}\theta_{j}B^{j}}{1-\sum_{i=1}^{p}\varphi_{i}B^{i}}a_{t}=\frac{\theta (B)}{\varphi (B)}a_{t}$$

According to Equation (4), the ARMA model describes a system with a transfer function of θ(B)/φ(B), where φ(B) represents the inherent characteristics of the system and θ(B) represents the relationship between the system and the external environment. As the input signals of large civil engineering structures are often difficult to test or measure, the performance and behavior of the structure can only be inferred through the analysis of output signals. The ARMA model can be established by analyzing the output signal of the structure, without considering the specific information of the input signals, and only using the white noise sequence $\left\{a_{t}\right\}$ as input to establish an analysis model. This makes the ARMA model increasingly widely used in the field of civil engineering. In the analysis of time series models, the AR, MA, and ARMA models have their own characteristics, which can be used to preliminarily determine the appropriate time series model. The characteristics of the models are shown in Table 1.

#### 3.1.2. Determination of ARMA Order

When applying the ARMA model for time series analysis, it is necessary to determine the order of the ARMA model, and the quality of the order setting has a significant impact on the parameter identification results. If the order is too high, the model will be too complex, resulting in overfitting and unnecessary calculations; if the order is too low, the model will be too simple to capture important features of the time series, leading to poor model fitting. Currently, one of the mostly used methods for determining the order of the ARMA model is AIC [22]. The function of AIC is defined as follows:

(5) $$AIC\left(p+q\right)=\ln\left(\sigma_{a}^{2}\right)+2(p+q)/N$$

In Equation (5), *N* represents the length of the time series $x_{t}$, $\sigma_{a}^{2}$ represents the variance of the ARMA model residuals, and *p* and *q* represent the AR and MA order, respectively. As the sum of model orders (p+q) increases, $\ln\left(\sigma_{a}^{2}\right)$ will decrease and 2(p+q)/N will increase. Therefore, under the given parameter estimation method, the order with the minimum $AIC\left(p+q\right)$ value should be chosen.

#### 3.1.3. Estimation of ARMA Parameter

This article used the long autoregressive (LAR) method to estimate the parameters of the ARMA model. Compared with other methods for parameter estimation, the LAR method can transform nonlinear regression problems into linear regression problems, which can simplify the calculation process and improve efficiency. According to the theory of LAR method, the ARMA model and the AR model are equivalent mathematical models. Therefore, the parameters of the AR model can be estimated first, and then the parameters of the ARMA model can be estimated based on the relationship of the transfer functions. The equivalent relationship between the transfer functions of AR and ARMA models is as follows:

(6) $$\frac{1}{1-\sum_{i=1}^{p}I_{i}B^{i}}=\frac{1-\sum_{j=1}^{m}\theta_{j}B^{j}}{1-\sum_{k=1}^{n}\varphi_{i}B^{k}}$$

In Equation (6), the left side represents the transfer function of an AR(*p*) model, and the inverse function $I_{i}$ is equal to the parameter $\varphi_{i}$ of the AR model. The right side represents the transfer function of an ARMA(*n*,*m*) model. By moving the same power coefficients of the shift operator *B* in Equation (6), it can be deduced that

(7) $$\begin{array}{c} \varphi_{1}=\theta_{1}+I_{1}\\ \varphi_{2}=\theta_{2}-\theta_{1}I_{1}+I_{2}\\ \ldots \ldots \\ \varphi_{n}=\theta_{m}I_{n-m}-\ldots -\theta_{2}I_{n-2}-\theta_{1}I_{n-1}+I_{n}\\ 0=\theta_{m}I_{k-m}-\ldots -\theta_{2}I_{k-2}-\theta_{1}I_{k-1}+I_{k} \end{array}$$

According to Equation (7), the solution for $\varphi_{i}(i=1,2,\ldots ,n)$ can be obtained as follows:

(8) $$\begin{array}{c} \left[\begin{array}{c} \varphi_{1}\\ \varphi_{2}\\ \varphi_{3}\\ \vdots \\ \varphi_{n} \end{array}\right]=\left[\begin{array}{c} \theta_{1}\\ \theta_{2}\\ \theta_{3}\\ \vdots \\ \theta_{n} \end{array}\right]+\left[\begin{array}{ccccc} 1 & 0 & 0 & \ldots & 0\\ -\theta_{1} & 1 & 0 & \ldots & 0\\ -\theta_{2} & -\theta_{1} & 1 & \ldots & 0\\ \vdots & \vdots & \vdots & \ddots & \vdots \\ -\theta_{n-1} & -\theta_{1} & -\theta_{1} & \ldots & 1 \end{array}\right]\left[\begin{array}{c} I_{1}\\ I_{2}\\ I_{3}\\ \vdots \\ I_{n} \end{array}\right]\\ (\mathrm{when}\;j>0,\;\theta_{j}=0)\; \end{array}$$

For the last term in Equation (7), let k=n+1,n+2,…,n+m, where n+m=p. Then, $I_{i}(i=n+1,n+2,\ldots ,n+m)$ can be expressed as

(9) $$\left[\begin{array}{c} I_{n+1}\\ I_{n+2}\\ I_{n+3}\\ \vdots \\ I_{n+m} \end{array}\right]=\left[\begin{array}{ccccc} I_{n} & I_{n-1} & I_{n-2} & \ldots & I_{n+1-m}\\ I_{n+1} & I_{n} & I_{n-1} & \ldots & I_{n+2-m}\\ I_{n+2} & I_{n+1} & I_{n} & \ldots & I_{n+3-m}\\ \vdots & \vdots & \vdots & \ddots & \vdots \\ I_{n+m-1} & I_{n+m-2} & I_{n+m-3} & \ldots & I_{n} \end{array}\right]\left[\begin{array}{c} \theta_{1}\\ \theta_{2}\\ \theta_{3}\\ \vdots \\ \theta_{n} \end{array}\right]$$

Both Equations (8) and (9) are linear equation sets about $\theta_{j}$, so $\theta_{j}$ can be solved first according to Equation (9), and then $\varphi_{i}$ can be solved according to Equation (8).

Based on the theoretical research of Ljung [23], this paper adopted the order scheme of p=2m, q=2m−1 to establish the ARMA model of a multi-degree-of-freedom system, which is also a widely used model scheme in engineering [24].

#### 3.1.4. Principal Component Analysis

There is a large amount of environmental noise in the on-site measurement data of a structure, which greatly reduces the efficiency and reliability of damage monitoring. Therefore, it is necessary to conduct feature analysis on the data to remove the noise influence and amplify the impact of structural damage on data changes. In this paper, PCA was adopted for feature extraction, which can filter out noise and redundancy in the original high-dimensional feature space data and transform it into interpretable data in a low-dimensional feature space. It can eliminate confusing data while preserving the main information [25].

For a dataset $X\in R^{n\times p}$ with *p* variables and *n* samples,

(10) $$X=\left[\begin{array}{cccc} x_{11} & x_{12} & \ldots & x_{1p}\\ x_{21} & x_{22} & \ldots & x_{2p}\\ \vdots & \vdots & \ddots & \vdots \\ x_{n1} & x_{n2} & \ldots & x_{np} \end{array}\right]=\left[\begin{array}{cccc} X_{1} & X_{2} & \ldots & X_{p} \end{array}\right]$$

the *i*-th principal component of *X* can be represented as

(11) $$\begin{array}{c} Y_{i}=u_{1i}X_{1}+u_{2i}X_{2}+\cdots +u_{pi}X_{p}\\ \left(i=1,\;2,\;\cdots ,\;p\right) \end{array}$$

where $u_{pi}$ represents the characteristic vector. After standardizing the original data matrix *X*, the resulting matrix X* has a covariance matrix *S* that is equal to the correlation coefficient matrix:

(12) $$S=\frac{1}{n-1}X^{*}X^{*T}$$

The eigenvalues of the covariance matrix of X* are denoted by $\lambda_{1},\;\lambda_{2},\;\ldots ,\lambda_{p}$, and $\lambda_{1}\geq \lambda_{2}\geq \ldots \geq \lambda_{p}>0$. The number of principal components *n* is determined by the variance accumulative contribution rate (ACR) $\sum_{i=1}^{n}\lambda_{i}/\sum_{i=1}^{p}\lambda_{i}$. If the ACR of the first *n* principal components is not less than 85%, it indicates that the first *n* principal components have incorporated most of the original data information [26].

### 3.2. Damage Sensitive Feature

DSF refers to the characteristic or indicators used to identify and locate potential structural damage, which can reflect the internal state changes of the structure or system. It requires the establishment of an ARMA model to analyze the signal, and the parameters of the model include the inherent characteristics of the system. By extracting the carrier containing structural features, it can be determined whether the structure is damaged. Because the AR coefficients reflect the dynamic characteristics of the system, they can be used to construct the DSF.

#### 3.2.1. Mahalanobis Distance

The principle of the structural damage detection method based on statistical pattern recognition is to compare two sets of model parameters, one set is the model feature parameters of the structure in the healthy state, and the other set is the feature parameters in the unknown (to be identified) state. In the damage identification method based on the ARMA model, the MD is usually used to determine the distance between the sample data and the reference data after feature extraction, and then evaluate the health status of the structure. MD calculates the distance between two multivariate sets by considering the correlation between them, which is an effective method for computing the similarity between two unknown-state sample sets. The expression of the MD between the sample set *z* and the population *G* is:

(13) $$d_{M}(z,G)={\left[\left(z-v_{G}{)}^{T}\sum^{-1}(z-v_{G}\right)\right]}^{\frac{1}{2}}$$

where $v_{G}$ represents the mean vector of *G*, and Σ represents the covariance matrix of *G*.

#### 3.2.2. Construction of DSF

ARMA models were established for the unknown-state sample set, reference sample set, and training sample set. The DSF was constructed by measuring the differences between the data parameters of the ARMA model under the unknown state and the healthy state. The formula is as follows:

(14) $$DSF(u,r)=\frac{d_{M}^{2}(u,T)}{d_{M}^{2}(r,T)}$$

where *u* represents the unknown-state sample set, *r* represents the reference sample set of the healthy state, and *T* represents the training sample set of the healthy state. A DSF value close to 1 indicates that the structure is in a healthy state, while a DSF value greater than 1 indicates damage. Moreover, the severity of the damage is positively correlated with the magnitude of the DSF.

## 4. Results and Discussions

This section provides an explanation and discussion of the damage identification results using the FE models and SHM measured data. Firstly, a concise introduction is provided for the FE model, which includes the setup of five simulated DCs. The establishment of a time series model and the detailed results of damage identification are then elaborated. Finally, the obtained results are thoroughly explained and discussed. Furthermore, this section is based on two years of SHM data collected from the timber lattice shell dome of Taiyuan Botanical Garden. The proposed damage identification method from this article is applied to identify the damage in the dome, and the identification results are subsequently analyzed and discussed.

### 4.1. Damage Identification Based on Numerical Models

Structural damage in practical engineering exhibits characteristics such as randomness and finiteness, making it difficult to determine the effectiveness and availability of time series models through measured data. However, FE methods can be used to simulate the health and damage status of the structure, in order to establish and validate the ARMA model.

To verify the feasibility and applicability of the ARMA model for large-span timber lattice structures, this paper used Midas Gen, a general structural analysis software, to perform FE modeling and analysis for the timber lattice dome of Taiyuan Botanical Garden. The stiffness of individual or multiple components was reduced to simulate the structural damage conditions (DC). Gaussian white noise was used to excite the structure, and the response results of multiple measuring points (MP) were extracted to evaluate the damage identification effect of the ARMA model.

#### 4.1.1. Finite Element Model

**Figure 2:**
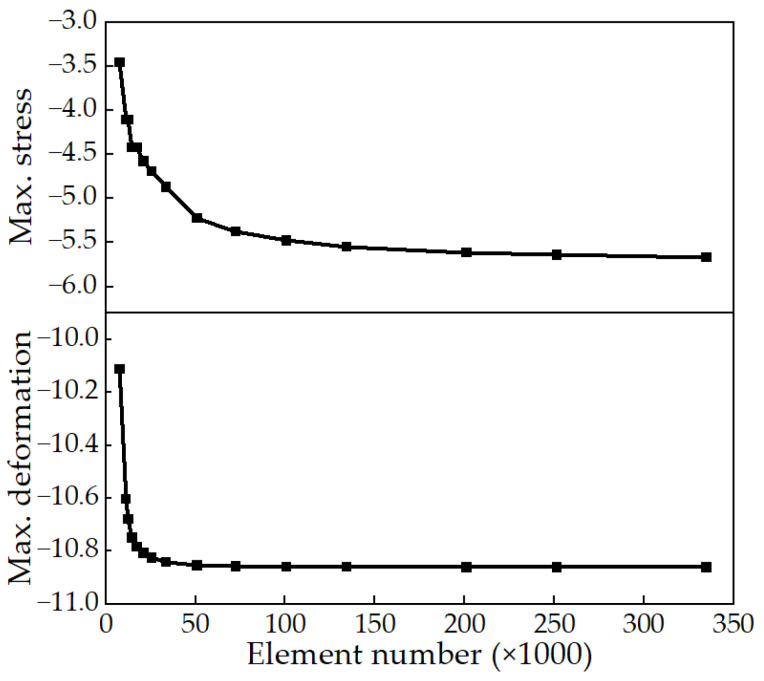
Mesh convergence analysis.

This paper used 3D line elements to analyze the spatial structure of the dome. The timber beams were modeled with beam elements, while the cable net was modeled with cable (tensile only) elements. In this study, the maximum main stress and maximum vertical deformation were employed as the convergence indicators. The analysis findings demonstrated that as the number of elements increased from 7732 to 334,817, both the maximum stress and maximum deformation exhibited a gradual convergence. The mesh convergence plot is shown in Figure 2. Notably, the maximum stress was more sensitive to the element number, thus serving as the primary controlling factor. Taking into account both computational accuracy and efficiency, the 134,372-element model was ultimately selected, which achieved a maximum stress convergence within a 5% range and satisfied the required computational accuracy.

**Figure 3:**
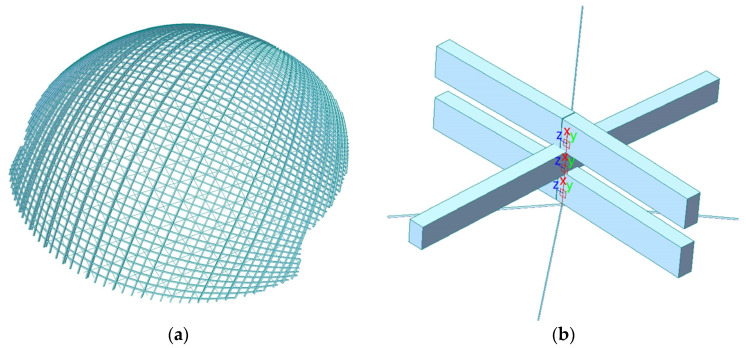
FE model. (**a**) Overall structure; (**b**) timber beam’s stacking joint.

**Figure 4:**
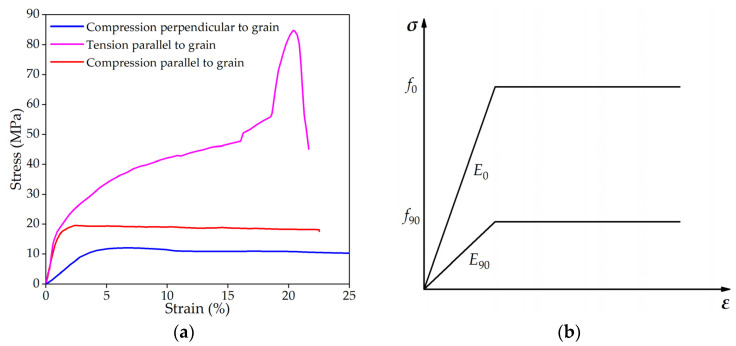
Material properties. (**a**) Material test data (GL28h), reprinted with permission from Ref. [27]. 2022, Shuizhong Jia; (**b**) bilinear material model of glued laminated timber [31].

The FE model took into account the fact that the three-layer timber beams are not coplanar, as shown in Figure 3. The timber beams are made of European spruce glued laminated timber GL28h, with a main beam section of 200 mm × 400 mm (width × height) and a secondary beam section of 200 mm × 300 mm (width × height). The components of the lattice structure are mainly subjected to axial forces, and glued laminated timber has relatively ideal material properties. According to material tests [27] and related studies [28,29,30], the FE material model adopted an orthotropic bilinear elastic-plastic model. The material test data and FE material model were shown in Figure 4, and the material properties are detailed in Table 2. The cable has a diameter of 26 mm, and the material used is austenitic steel 06Cr17Ni12Mo2 (316 stainless steel), with an elastic modulus of 160,000 MPa and a design pre-tension value of 40 kN.

Based on the measured data of SHM, simple modifications were attempted on the FE model. Initially, the acceleration signal during the early stage of structural service was processed and analyzed. The time-domain data were then transformed into frequency-domain data using Fourier transform. To eliminate high-frequency noise, a low-pass filter of 50 Hz was applied. The power spectral density (PSD) of the acceleration data was calculated, and the dominant frequency with significant amplitude in the signal was extracted through peak detection. This allowed for the determination of the natural frequency of the structure.

Subsequently, modifications were made to the FE model parameters. The elastic modulus and density of the material were adjusted, along with the magnitude of the additional constant load (converted into mass) on the roof, and the connection stiffness of the joints. These modifications aimed to align the natural vibration characteristics of the FE model with the analysis results obtained from the measured data.

#### 4.1.2. Damage Conditions

When establishing a time series model, the first step is to determine the sampling time interval Δt and the sample length *L* for the continuous signal. When sampling a continuous signal, a reasonable sampling time interval is $\Delta t<1/(2f_{max})$, and a reasonable sample length is $L>1/\Delta f_{min}$, where $f_{max}$ is the highest frequency in the interested vibration modes and $\Delta f_{min}$ is the minimum difference between adjacent frequencies [32].

**Figure 5:**
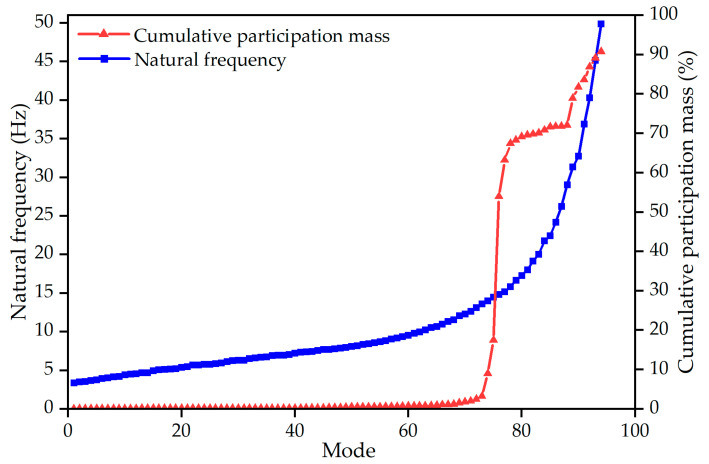
100-order Z-axis modal analysis.

Given the complex natural vibration characteristics of the lattice shell structure, a 100-order natural vibration modal analysis was conducted for the FE model. The results of the modal analysis are presented in Figure 5 and Table 3. The analysis revealed that the natural frequencies of the lower order modes are closely spaced, with a difference of only 8.92 Hz between the first and 70th modes. By the 94th mode, 90% of the cumulative participation mass of the vibration modes was reached, indicating that the first 94 modes could capture the majority of the natural vibration characteristics of the structure. Therefore, the appropriate sampling frequency and sample length were calculated based on the first 94 modes. The maximum frequency in the first 94 modes was approximately 49.85 Hz, and the minimum difference between adjacent frequencies was 0.0109 Hz. According to the sampling theories [32], the sampling time interval of this structure should be less than 0.0101 s, and the sample length should be greater than 91.75 s. Therefore, a sampling frequency of 100 Hz and a sample length of 120 s were adopted to establish a time series model. According to the basic principle of time series analysis, the signal input needs to meet the characteristics of stationary, normal, and zero-mean, and Gaussian white noise can just meet the above characteristics. Thus, Gaussian white noise generated by MATLAB was used as the environmental excitation to conduct the time history analysis on the FE model.

**Figure 6:**
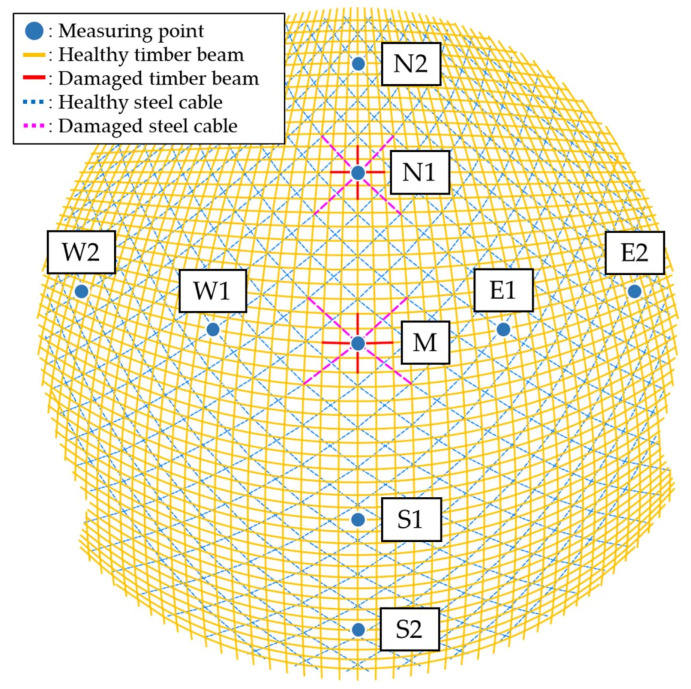
Distribution of MPs and damaged components.

In practical engineering, vibration sensors were used for SHM of the dome structure. Specifically, nine vibration MPs were set up at the joint positions, taking into account the shape and characteristics of the structure. From above, the MPs were distributed at the central point of the dome and in the directions of east, west, south, and north. In the vertical direction, the MPs were evenly distributed at the top, middle, and bottom of the structure. During the time history analysis on the FE models, acceleration response data were extracted from the nine MPs. The layout of the MPs was shown in Figure 6. In the FE analysis process, stiffness reduction was applied to local timber beam and steel cable elements to simulate damage or stiffness degradation of the components. This paper developed five sets of DCs, including damage to single and multiple components. The five simulated DCs are detailed in Table 4.

#### 4.1.3. Establishment of ARMA Model

This study established time series models for all nine MPs under various DCs as shown in Table 4. In this paper, the process of establishing a time series model was demonstrated using the acceleration time history data of MP M, which is located at the top of the dome structure.

**Figure 7:**
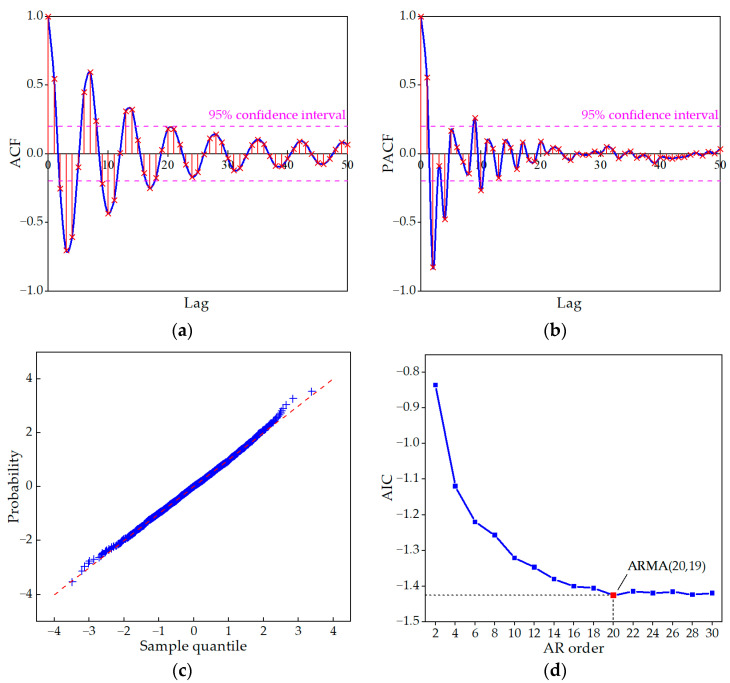
ARMA order selection. (**a**) ACF; (**b**) PACF; (**c**) normal distribution probability; (**d**) AIC of each order combination.

Before establishing the ARMA model, it is necessary to standardize the sample dataset. The normal distribution probability plot of the processed data, which was shown in Figure 7c, indicated that the processed sample is a stationary, normal, and zero-mean time series, satisfying the prerequisite for establishing a time series model. After preprocessing the data, the ACF and PACF of the data sample were calculated, as shown in Figure 7a,b. The ACF and PACF gradually decreased to within the 95% confidence interval, and both exhibited tailing characteristics. According to Table 1, an ARMA model could be established for the data sample.

Subsequently, the AIC and LAR method were used to determine the order and estimate the parameters of the model. Figure 7d shows the AIC results of the ARMA(2*m*,2*m*−1) model for the first 30 orders. Based on the AIC results, an ARMA(20,19) model was established for the MP M, and its estimated parameters are shown in Table 5.

#### 4.1.4. Damage Identification of FE Models

ARMA(*p*,*q*) models were established for the responses obtained from the FE models under the five DCs shown in Table 4. To alleviate the computational burden arising from the high AR order, dimensionality reduction could be achieved by conducting PCA on its parameters. Based on the principle of PCA, the AR parameters of each MP were analyzed for five distinct DCs.

Table 6 presents the eigenvalue and ACR obtained through PCA for the data of each MP. The eigenvalue quantifies the extent to which each principal component explains the variation in the data, while the ACR represents the proportion of total variance explained by the first *n* principal components. An ACR exceeding 85% indicates that the primary components already encompass most of the feature information [26]. Based on the PCA results, the eigenvalues of the first and second order AR coefficients for each MP were nearly 10 and 4, respectively. Furthermore, the ACRs for the first three orders at each MP all exceeded 90%, surpassing the 85% threshold. In some cases, the ACR even reached approximately 99%. Consequently, it can be inferred that employing the first three order AR coefficients to construct DSF can yield sufficiently accurate outcomes in theory.

**Figure 8:**
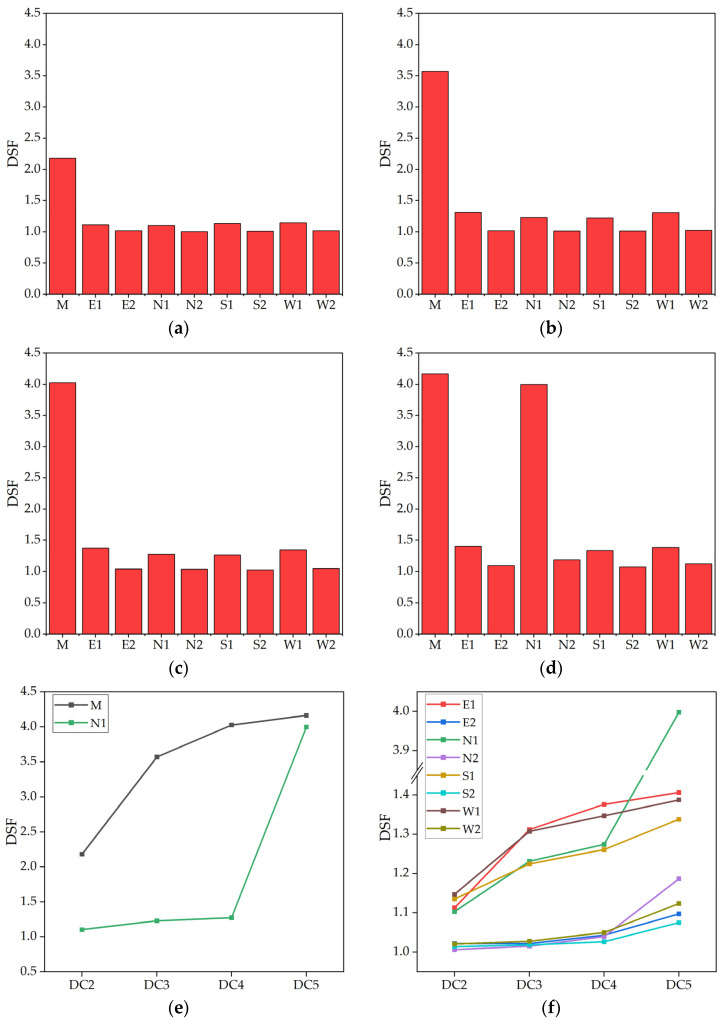
Damage identification results of FE models. (**a**) Results under DC 2; (**b**) results under DC 3; (**c**) results under DC 4; (**d**) results under DC 5; (**e**) results of M and N1; (**f**) results of undamaged MPs.

The acceleration time series data from each MP under the healthy state (DC 1) was subjected to windowing processing, resulting in two equally divided sections. The first 60 s of the resulting time series data, comprising 6000 sample data points, was utilized as training sample data, while the last 60 s, also comprising 6000 sample data points, was used as reference sample data. The remaining data obtained from other DCs were unknown-state sample data. The first three order AR coefficients of the ARMA model were extracted for each sample data. A principal component matrix was established and the MD was calculated between the principal component matrices of a single MP under different DCs. Corresponding DSF for damage identification was constructed according to Equation (14). The DSF for each MP is presented in Figure 8.

Based on the results of damage identification, the following observations could be made:

(1) When the damage of the timber beam at MP M was 40% (DC 2), the DSF at M was 2.1799, which was significantly higher than the DSF at other MPs, not exceeding 1.15. As the damage increased to 80% (DC 3), the DSF at M increased to 3.5720, while the average DSF at other MPs was 1.1450. This represented a 63.9% increase in DSF compared to the 40% damage level. When both the timber beam and steel cable at M were damaged simultaneously (DC 4), the DSF further increased to 4.0281, which was approximately 12.77% higher than the previous DC. The average DSF value at other MPs was 1.1773. These results demonstrate that the DSF constructed using PCA and MD is highly sensitive to component damage, capable of identifying and locating damage to timber beams and steel cables, and positively correlates with the degree of damage. Therefore, it could reflect the degree of damage to the damaged components to a certain extent.
(2) When the timber beam and steel cable at MPs M and N1 were simultaneously damaged by 80% (DC 5), the DSF of M and N1 were 4.1645 and 3.9983, respectively, which were significantly higher than the DSF values of other MPs, ranging from 1.07 to 1.38. This demonstrates that the proposed damage identification model can effectively identify conditions where multiple components are damaged simultaneously.
(3) As the damage at MP M intensified from DC 2 to 4, the DSF of M significantly increased, while the DSF of adjacent MPs N1, E1, S1, and W1 also slightly increased, with an average increase of approximately 0.19. However, the DSF of MPs N2, E2, S2, and W2 did not increase significantly, with an average increase of no more than 0.1. Under DC 5, further damage occurred at MP N1. During this process, the DSF of N2 and M, which were closest to N1, increased the most, with values of 0.2070 and 0.1364, respectively. However, the DSF of S2, W2, and E2, which were farthest from N1, remained almost unchanged, with values of 0.049, 0.074, and 0.054, respectively. This suggests that structural damage at a specific location can cause an increase in the DSF of surrounding MPs, and the degree of increase is inversely proportional to the distance. This phenomenon may be attributed to the changes in the local natural vibration characteristics caused by component damage, which were not significantly far from the damage location.
(4) Assuming that no MPs are set at M and N1, meaning that the DSF of M and N1 were not taken into account, under DC 4, the DSF values of E1, W1, and S1 experienced a significant increase, ranging from 1.25 to 1.35, while the DSF of the remaining MPs showed no noticeable changes. This suggests that the damage occurred in areas near E1, W1, and S1. It can be inferred that the damage may have occurred around M, which aligns with the severe damage observed at M under DC 4. From DC 4 to 5, the DSF of N2 increased significantly, while the DSF of the other MPs increased slightly and to a similar extent. This implies that the damage may have occurred in a location close to N2. As determined, N1 sustained damage during the transition from DC 4 to 5, which is consistent with this characteristic. The aforementioned statement indicates that even if the damage does not occur at MPs, the approximate location of the damage can be determined based on the DSF of the existing MPs, thereby achieving the objective of damage monitoring. This is advantageous for optimizing the number of MPs.

### 4.2. Damage Identification Based on SHM Data

**Figure 9:**
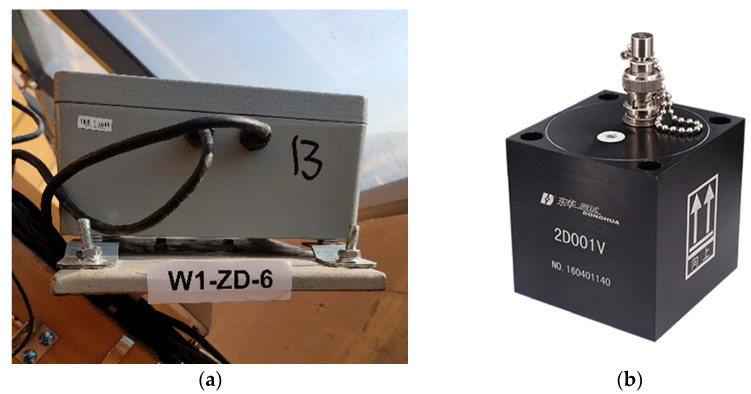
Data acquisition device. (**a**) Tri-dictional vibration monitoring module; (**b**) 2D001 magnetoelectric vibration sensor.

This paper presented a damage identification study of the dome structure at Taiyuan Botanical Garden, utilizing measured data from vibration sensors. The structure employed glued laminated timber beam as its primary load-bearing component, and exhibited a large span, complex natural vibration characteristics, and susceptibility to wind vibration, earthquakes, and other impacts. To ensure the structure’s safety, long-term SHM has been conducted since its service. Based on the structure’s characteristics and FE analysis results, nine vibration MPs shown in Figure 6 were selected to monitor the structure’s vibration behavior. These MPs were equipped with the 2D001 magnetoelectric vibration sensors produce by Donghua Testing Technology Co. Ltd. (Taizhou, China), with a maximum sampling frequency of 100 Hz. The data acquisition device is shown in Figure 9, and the technical parameters of the 2D001 sensor are shown in Table 7.

**Figure 10:**
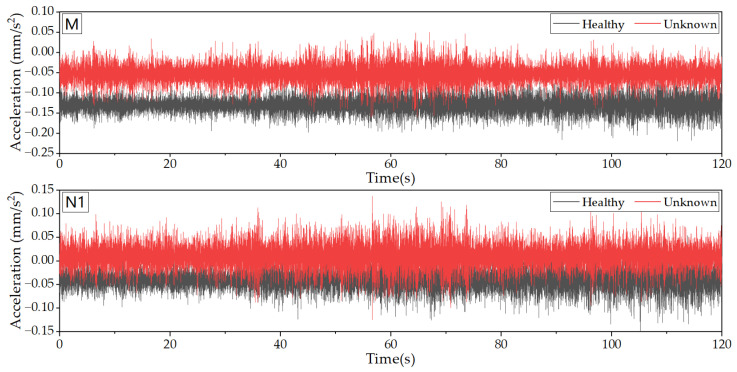
Original SHM data (M and N1).

Under normal working conditions, the only dynamic load on this structure was wind load. However, due to the fact that the dome is covered with glass, the structure was exposed to sunlight radiation during the day, resulting in temperature gradients on the surface that may have an uncertain impact on the sampling data and final analysis results. Therefore, this study selected measured data at night as the sample data. Specifically, acceleration data from the initial stage of structural service (October 2020) were chosen as the healthy-state sample data, while data after two years of service (October 2022) were selected as the unknown-state sample data, with a sample length of 120 s. Hence, ARMA modeling was performed and the health status of the structure after two years of service was evaluated. Figure 10 displays partial data for the initial stage and after two years of service.

The results of the calculation indicated that the ACF and PACF of the model are tailing, which satisfies the prerequisite for ARMA modeling. The AIC was employed to determine the order of the ARMA(2*m*,2*m*−1) model for the measured data. The findings reveal that the ARMA(28,27) model had the lowest AIC value of −1.4235 among the first 30 orders, indicating that this order scheme can achieve a high degree of fitting. The vertical acceleration data of nine vibration sensors at each MP were preprocessed, and ARMA(28,27) models were established. PCA was performed on the AR parameters using a set of measured data from each MP under both healthy and unknown states. The analysis results showed that the first 5 order AR parameters of any ARMA model contained 90% of the model’s damage characteristic values. Therefore, the first 5 order AR coefficients were utilized to construct subsequent DSF.

The acceleration time series of each MP in a healthy state was extracted and partitioned into two equal segments by windowing. The first 60 seconds’ 6000 sample data points were designated as training sample data, while the last 60 seconds’ 6000 sample data points were designated as reference sample data. The remaining data were used as unknown-state sample data. The MD was computed to generate DSF for damage identification.

**Figure 11:**
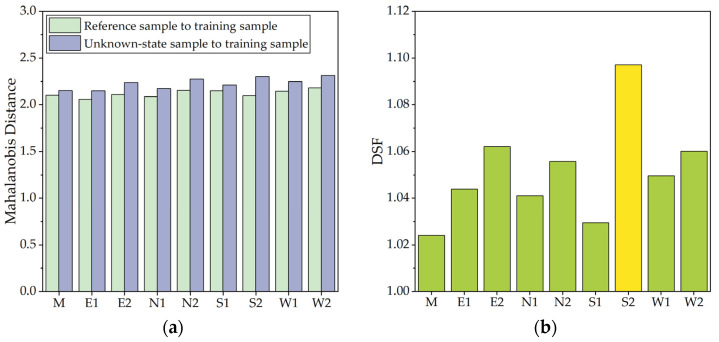
Damage identification results. (**a**) MD results; (**b**) DSF results.

**Figure 12:**
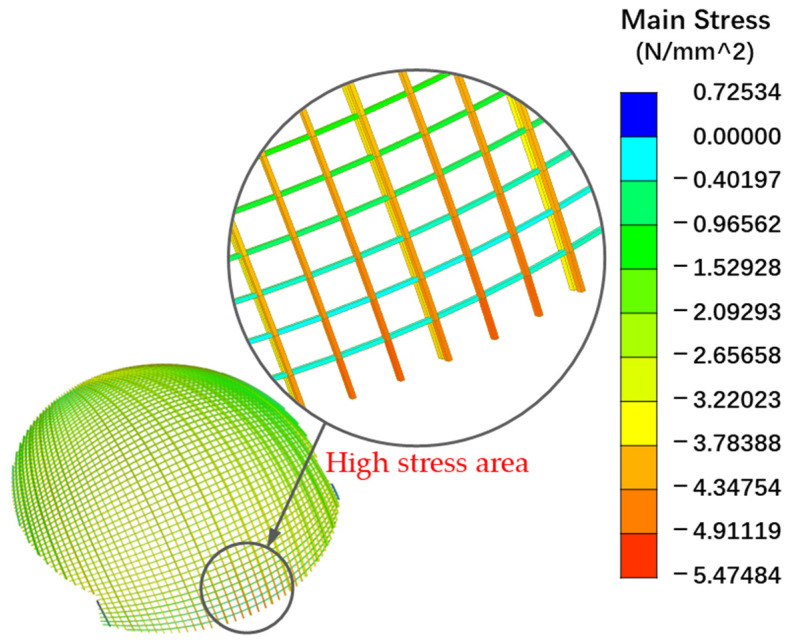
High stress area exhibited in static FE analysis result.

According to the analysis results, the MDs of each sample were not significantly different, and the DSF of all MPs were close to 1.0, indicating that the structure was almost not damaged after two years of service. The results of MD and DSF of each MP were shown in Figure 11. In addition, it could be found that the DSF of MP S2 was 1.097, while the maximum value of DSF of other MPs was 1.062, and the average value was 1.046, indicating that the damage degree of the component at S2 was slightly higher than that of other MPs. Figure 12 shows the static FE analysis results of the dome. Under the combined action of dead load and roof live load, the stress level of the timber components around S2 was higher than that in other areas. Moreover, real-time dynamic loads such as wind loads and temperature effects might have adverse effects on local areas. These adverse effects might be the potential factors leading to the increase of DSF at S2. Therefore, it is necessary to pay attention to the SHM data of this dome structure, and focus on the acceleration data of MP S2, so as to timely warn when the structure suffers serious damage.

## 5. Conclusions

Structural damage identification is a crucial analysis method in SHM, with significant scientific and engineering applications. To address the challenges of incomplete modal information, high modal density, and large degrees of freedom in large-span spatial lattice structures, this paper proposed a structural damage identification method based on the ARMA model and PCA method for structures of this kind. Based on the above research, the following conclusions can be drawn:

(1) This article was based on a time series model and introduced PCA for extracting principal components. In this study, only the first three AR coefficients were required to achieve damage identification and it demonstrated high accuracy.
(2) The MD is advantageous compared to other distance methods in constructing DSF because it considers the differences and correlations in the variability of variables for each observation. Additionally, it can effectively calculate the influence of scale on different measurement values.
(3) Verified by the FE model, the damage identification method proposed in this article was found to be sensitive to structural damage. It could, to a certain extent, accurately locate the site of structural damage and reflect the extent of the damage. Therefore, it can be effectively utilized for practical structural damage identification.
(4) Based on SHM data, the paper identified the damage of the structure after two years of service. The results indicated that the structure is in a relatively healthy state, but the DSF of MP S2 is slightly higher than those of other MPs. In the future, it is necessary to pay attention and give timely warnings if necessary.

However, there are still some shortcomings in this article. The next step should be to conduct more in-depth research on the rationality of the number and distribution of measurement points, as well as the refinement of the FE model correction.

## Figures and Tables

**Table 1:** Basic characteristics of time series models.

Model Type	Autocorrelation Function (ACF)	Partial Autocorrelation Function (PACF)
AR	Tailing	Truncation
MA	Truncation	Tailing
ARMA	Tailing	Tailing

**Table 2:** Material property of GL28h.

*E*_c,0_ (MPa)	*E*_t,0_ (MPa)	*E*_c,90_ (MPa)	*f*_c,0_ (MPa)	*f*_t,0_ (MPa)	*f*_c,90_ (MPa)
13,901	7438	185	29.6	77.5	5.0

**Table 3:** Natural frequencies of the FE model.

Mode Order	Frequency (Hz)	Mode Order	Frequency (Hz)	Mode Order	Frequency (Hz)	Mode Order	Frequency (Hz)
1	3.3537	26	5.8696	51	8.2039	76	14.8373
2	3.5002	27	5.9719	52	8.3625	77	15.1701
3	3.5525	28	6.134	53	8.4575	78	15.8307
4	3.6825	29	6.2517	54	8.5635	79	16.6591
5	3.7451	30	6.2953	55	8.7213	80	17.2487
6	3.9603	31	6.3233	56	8.847	81	17.9974
7	4.0067	32	6.5079	57	9.0585	82	19.1284
8	4.1308	33	6.6175	58	9.1913	83	20.0353
9	4.2118	34	6.6899	59	9.3714	84	21.7652
10	4.431	35	6.7379	60	9.5392	85	22.4354
11	4.4916	36	6.9291	61	9.7873	86	24.1665
12	4.5736	37	6.9573	62	9.9823	87	26.2336
13	4.6526	38	6.9682	63	10.2407	88	29.05
14	4.6659	39	7.0649	64	10.5235	89	31.3453
15	4.9154	40	7.2344	65	10.6672	90	32.7382
16	5.0791	41	7.3614	66	10.9764	91	36.9209
17	5.1145	42	7.3811	67	11.3259	92	40.3238
18	5.1646	43	7.4171	68	11.5494	93	45.1509
19	5.2276	44	7.5568	69	12.0646	94	49.8536
20	5.3595	45	7.6886	70	12.2717	95	59.3389
21	5.4691	46	7.7193	71	12.6162	96	72.188
22	5.6584	47	7.7773	72	13.0992	97	88.9345
23	5.6804	48	7.8668	73	13.6149	98	121.4361
24	5.7494	49	7.9797	74	13.9864	99	180.6925
25	5.7668	50	8.1043	75	14.4609	100	349.0827

**Table 4:** Damage conditions.

Condition No.	MP M		MP N1	
	Beam	Cable	Beam	Cable
1	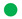	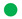	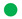	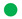
2	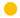	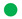	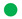	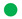
3	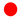	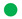	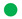	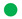
4	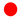	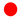	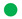	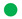
5	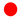	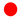	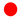	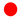

Note: 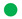: in a healthy state; 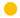: damaged by 40%; 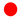: damaged by 80%.

**Table 5:** Estimated ARMA model parameters.

Order	AR Coef.	MA Coef.	Order	AR Coef.	MA Coef.
1	0.1956	0.9294	11	0.0487	−0.3235
2	−0.3641	0.1359	12	−0.0731	−0.1088
3	−0.0754	−0.3965	13	−0.0082	0.3625
4	−0.2056	−0.4014	14	−0.4730	0.8166
5	−0.1359	−0.0875	15	0.1008	0.4343
6	−0.1749	0.1776	16	−0.2690	−0.0291
7	0.2484	0.4023	17	−0.1583	−0.1125
8	−0.1414	0.1495	18	−0.3139	0.0210
9	0.0141	−0.2229	19	0.0208	0.1027
10	0.0242	−0.2021	20	0.0635	-

**Table 6:** PCA results.

**Principal** **Component**	**M**		**E1**		**E2**	
	**Eigenvalue**	**ACR**	**Eigenvalue**	**ACR**	**Eigenvalue**	**ACR**
1	10.3245	51.62%	14.4691	72.35%	13.7320	68.66%
2	4.6304	74.77%	3.2225	88.46%	3.1710	84.52%
3	3.8133	93.84%	1.2549	94.73%	1.9219	94.12%
4	0.8551	98.12%	1.0002	99.73%	1.0343	99.30%
5	0.3768	100.00%	0.0532	100.00%	0.1407	100.00%
**Principal** **Component**	**W1**		**W2**		**S1**	
	**Eigenvalue**	**ACR**	**Eigenvalue**	**ACR**	**Eigenvalue**	**ACR**
1	15.0565	75.28%	10.4912	52.46%	12.8791	64.40%
2	4.0753	95.66%	6.4063	84.49%	4.0294	84.54%
3	0.7505	99.41%	1.8273	93.62%	2.8699	98.89%
4	0.1117	99.97%	1.0842	99.05%	0.2155	99.97%
5	0.0060	100.00%	0.1909	100.00%	0.0061	100.00%
**Principal** **Component**	**S2**		**N1**		**N2**	
	**Eigenvalue**	**ACR**	**Eigenvalue**	**ACR**	**Eigenvalue**	**ACR**
1	10.8759	54.38%	8.3965	41.98%	11.1385	55.69%
2	4.3566	76.16%	7.4009	78.99%	7.1796	91.59%
3	2.7723	90.02%	2.4547	91.26%	1.1040	97.11%
4	1.9425	99.74%	1.2340	97.43%	0.4086	99.15%
5	0.0527	100.00%	0.5139	100.00%	0.1693	100.00%

**Table 7:** Technical parameters of the 2D001 magnetoelectric vibration sensor.

Mode	0	1	2	3
Parameter	Acceleration	Low Velocity	Medium Velocity	High Velocity
Sensitivity	0.3 V/m·s^−2^	20 V/m·s^−1^	5 V/m·s^−1^	0.3 V/m·s^−1^
Capacity	20 m·s^−2^	0.125 m·s^−1^	0.3 m·s^−1^	0.6 m·s^−1^
Bandwidth (−3–+1 dB)	(0.25–100) Hz	(1–100) Hz	(0.5–100) Hz	(0.17–80) Hz
Output Impedance	50 kΩ			
Working Temperature	(−20–80) °C			
Dimensions	63 mm × 63 mm × 63 mm			
Weight	0.6 kg			

## Data Availability

The data presented in this study are available from the first author, (wangguoqing0066@link.tyut.edu.cn), upon reasonable request.
